# The Context, Process, and Outcome Evaluation Model for Organisational Health Interventions

**DOI:** 10.1155/2015/414832

**Published:** 2015-10-18

**Authors:** Annemarie Fridrich, Gregor J. Jenny, Georg F. Bauer

**Affiliations:** Division of Public & Organizational Health, Epidemiology, Biostatistics and Prevention Institute, University of Zurich, Hirschengraben 84, 8001 Zurich, Switzerland

## Abstract

To facilitate evaluation of complex, organisational health interventions (OHIs), this paper aims at developing a context, process, and outcome (CPO) evaluation model. It builds on previous model developments in the field and advances them by clearly defining and relating generic evaluation categories for OHIs. Context is defined as the underlying frame that influences and is influenced by an OHI. It is further differentiated into the omnibus and discrete contexts. Process is differentiated into the implementation process, as the time-limited enactment of the original intervention plan, and the change process of individual and collective dynamics triggered by the implementation process. These processes lead to proximate, intermediate, and distal outcomes, as all results of the change process that are meaningful for various stakeholders. Research questions that might guide the evaluation of an OHI according to the CPO categories and a list of concrete themes/indicators and methods/sources applied within the evaluation of an OHI project at a hospital in Switzerland illustrate the model's applicability in structuring evaluations of complex OHIs. In conclusion, the model supplies a common language and a shared mental model for improving communication between researchers and company members and will improve the comparability and aggregation of evaluation study results.

## 1. Introduction

Recent years have seen a rise of work-related health interventions targeting the entire organisation as complex social systems, thus referred to as organisational health interventions (OHIs) [1]. This paper refers to comprehensive OHIs, which are usually comprised of a mix of individual-directed interventions targeting employee stress management capacities and leadership behaviour combined with work-directed interventions, such as collective workshops targeting working conditions and social relations. Such interventions are portrayed as intricate change processes in complex social systems where implementation and outcomes are never a priori predictable [2, 3].

Currently available meta-analyses and reviews of evaluation studies reveal mixed results for the effectiveness of comprehensive OHIs [4, 5]. Thus, systematic evaluations of OHIs are considered to be ultimately needed, to understand intended and unintended and desirable and undesirable changes and moreover to pass on process and outcome knowledge to other researchers and practitioners in the field [2, 3, 6]. However, since OHIs vary in content, composition, context, targeted audience, and the desired outcomes of change, it is difficult to develop generally applicable evaluation principles [7]. A variety of different evaluation approaches for OHIs have evolved, which either focus on specific aspects of interventions to be evaluated or present general rationales to be further specified and operationalized in regard to a specific project [2, 7, 8]. Additionally, these approaches make use of a diverse terminology, all of which provide a challenge for practitioners and intervention researchers to integrate findings across studies and to select an appropriate evaluation approach for their own intervention project.

So far, only a few researchers have published generic models for the evaluation of complex OHIs. Some models focus on one evaluation category such as process evaluation [7], others are limited to special types of interventions such as preventive occupational health and safety interventions [9], and still others are rather applicable to single component interventions than to multicomponent interventions [10]. Many of them display context as the underlying frame of an intervention but limit it to its hindering or facilitating role [7, 8, 10]. Finally, most of the existing models and frameworks [8, 9] classify effectiveness research/effect evaluation as an autonomous phase after the intervention instead of considering it as a process of continuous observation and assessment throughout the intervention.

All these models and frameworks demonstrate the considerable effort that has been made to standardise and improve the evaluation of organisational health interventions. However, to make the results revealed by evaluations of comprehensive interventions more comparable, a model with clearly defined evaluation categories is needed. Such a model has to be applicable to different intervention types, like single component and multicomponent interventions, interventions that address psychosocial or physiological aspects, and those that focus on the individual, the organisation, or both. Thus, it must be general enough to cover all these different intervention types but at the same time concrete enough to distinguish between the context, process, and outcome aspects at different organisational levels in different interventions phases.

Furthermore, the model has to consider context not only as a static and confounding factor that hinders or facilitates the implementation process, but also as a transformable and essential part of the intervention. The model should also consider the outcome evaluation as a continuous process rather than as a particular, time-limited intervention phase. Moreover, it should be accompanied by examples and research questions that help intervention researchers conduct context, process, and outcome evaluations.

All of the aforementioned models and frameworks only partially meet these needs. Thus, this paper aims at developing a model defining and relating generic evaluation categories for OHIs. First, we develop, define, and relate the generic categories and subcategories of the model. Second, concepts to specify the main categories for developing measurable indicators are compiled, considering different intervention approaches and principles from the literature. Third, research questions that might guide the evaluation of an OHI according to the CPO categories and a list of concrete themes/indicators and methods/sources applied within the evaluation of an OHI at a hospital in Switzerland illustrate the model's applicability in structuring evaluations of comprehensive OHIs.

## 2. Overview of the Model

**Figure 1:**
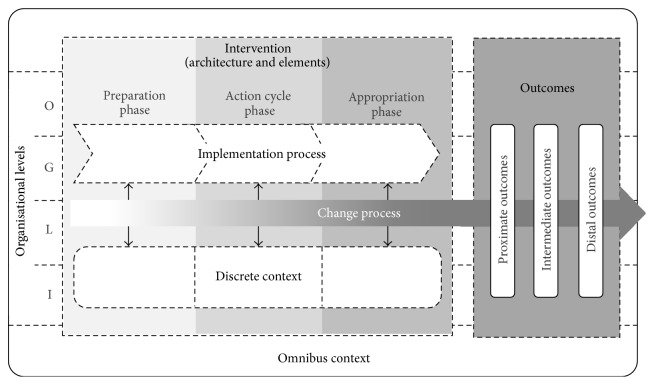
The context, process, and outcome (CPO) evaluation model. O: organisation; G: group; L: leader; I: individual.

The present model differentiates between the three categories context, process, and outcome evaluation of organisational health interventions and is thus labelled CPO evaluation model (see Figure 1). Context is seen as the underlying frame within an organisational health intervention is implemented, change occurs, and outcomes emerge. We further distinguish between the omnibus context which refers to the general intervention and implementation setting and the discrete context which refers to specific situational variables. In regard to the category of process, two subcategories are differentiated: the implementation process as the time-limited enactment of all steps and elements of the original intervention plan and the triggered change process as all the intended and unintended and observable and nonobservable mechanisms of alteration in the intervention context. This leads to outcomes, defined as all results of the change process observable and measurable in the intervention context. According to the phase of the change process, alterations of proximate, intermediate, and long-term outcomes can be distinguished.

Another important aspect of the model is its grid of phases and levels applied to these categories and subcategories. Whereas phases refer to temporal distinctions within the implementation process and its discrete context (preparation, action cycle, and appropriation), the levels refer to hierarchical aspects of the context and the intended intervention outcomes, spanning from the individual to the organisation. The following description starts with this overlaying grid of phases and levels and then proceeds to the categories and subcategories of context, process, and outcome.

## 3. Intervention Phases and Levels

### 3.1. Intervention Phases

OHIs usually consist of several elements such as participatory workshops, survey feedbacks, and information events. These planned intervention elements are comprised within overall intervention architecture, that is, the combination and sequence of intervention elements. Furthermore, intervention planning and evaluation concepts usually distinguish between three and five intervention phases [8, 9, 11–13]. They share in common the notion that an analysis is needed before actions can be planned and implemented. To reduce complexity, the CPO evaluation model proposes three intervention phases: the preparation phase, the action cycle phase, and the appropriation phase.

The first, so-called preparation phase comprises all activities needed to fit the intervention to the specific context and to obtain the commitment of the organisation for the following phases. This includes, for example, presentations and workshops with decision makers, a qualitative analysis of the intervention context, the planning of the intervention architecture (who is involved, when, and how in the OHI), the establishment of a steering group, and project leader.

The second phase is referred to as the action cycle phase that comprises all activities needed to trigger a change process that will improve organisational health on a broad scale. It encompasses the subphases analysis, action planning, enactment, and monitoring. Analysis, for example, an organisation-wide stress assessment, is considered part of the action cycle phase as it not only serves to generate information but also is an active intervention element since its mere implementation might trigger small changes, such as increased awareness, readiness for change, or sensibility for stressful issues [11]. It should be noted that we use the term enactment to replace the commonly used term implementation [8, 9, 12], as the activities in the preparation phase and the final appropriation phase should also be considered as part of the implementation process.

The third phase is named appropriation phase and comprises all activities needed to ensure the continuation, advancement, and diffusion of the change process triggered by the previous two phases. This phase refers to the period when intervention implementers and researchers usually have left the organisation. At that time, capacities for self-optimization have been built up and the organisation and its members have to take over the responsibility for the continuation of the triggered change processes, for example, in the form of continued, repeated action cycles and optimisation processes. As appropriation is a precondition for achieving sustaining long-term effects, we consider it a crucial element of any OHI evaluation.

### 3.2. Intervention Levels

OHI aims to impact different levels of an organisation, often referred to as combined, individual-organisational or multilevel interventions [14]. In this regard, the CPO evaluation model distinguishes the levels of individuals/leaders and groups/organisation (cf. the IGLO-levels by Nielsen and colleagues [15]). Thus, outcome evaluation should be conducted in consideration of these levels in order to make differentiated statements concerning the effectiveness of an intervention. This differentiation is also important for the evaluation of the intervention context, where different levels can be of importance during different phases. For example, during the preparation phase, organisational level factors such as strategic goals in regard to employee health are of particular importance; during the action cycle phase, leadership level factors such as line manager attitudes are crucial; during the appropriation phase, group level factors such as team climate and capacities for continued optimisation are relevant.

## 4. The Main Categories and Subcategories of the Model

### 4.1. Context

For a long time, researchers have considered context to be crucial for understanding the causes of success and failure of interventions [16]. Context is often considered as a process indicator [16–18], but in recent years, the perceived importance of and the attention to contextual issues have increased. Slowly but surely, context has dissociated itself from process issues, becoming an autonomous and meaningful factor in intervention research [19]. Research findings demonstrate that context is a very complex and intervention-specific factor [20] and that its effects can vary from subtle to powerful [21]. For instance, it might occur that an intervention aiming to improve individual resources is implemented in two teams of different sizes, and outcome evaluation may reveal the intervention to be effective in the smaller team while ineffective in the larger team. In the case that evaluators ignore further contextual aspects, they might conclude that the intervention works only in small teams. However, a more differentiated context evaluation might reveal that the line manager of the smaller team strongly supported the intervention while the line manager of the larger team tended to be critical of the intervention. This shows that neglect of contextual aspects could lead to fundamental fallacies concerning the effectiveness of interventions. Accordingly, the concept of context is broadly discussed in OHI research [2] and more broadly in organisational behaviour [21–23]. Various researchers treat context as an unspecific setting parameter that concerns environmental and situational aspects and limit its conceptualization to its facilitating and hindering functions [7, 18, 21]. Moreover, the context of an intervention is often considered as an undesired, uncontrollable, and unmanageable constraint that could be neither predicted nor controlled. For instance, Kompier and Kristensen [24] state that “interventions always take place in context, and that this context is not under control of scientists.”

The conceptualisation of context in the CPO evaluation model follows a taxonomy proposed by Bauer and Jenny [1] (in reference to human resource management practices distinguished by Delery and Doty [25]): “… the organization as the context of [organisational health] interventions might be considered for selecting and targeting the intervention [universalistic approach], for adapting the intervention to this context [contingency approach], or as the final target and actor of change [configurational approach].” Considering this reciprocal, transformational relationship between context and intervention, the CPO evaluation model defines context as the underlying frame that influences and is influenced by an organisational health intervention. As such, context is more than a static, nonchangeable boundary condition; it is a malleable riverbed directing the river of change and simultaneously being shaped by the river. When we consider context as a static boundary condition, we must accept the critical line manager in the above example as a nonchangeable hindering factor and run the risk of implementation failure. When we, on the other hand, consider context as an alterable condition, we can initially adapt the intervention (river) to the critical line manager (river bed) by assigning him/her a specific role, for example, by explicitly representing the critical perspective in the steering committee of the project. By this active involvement, his/her critical position might be transformed into one of constructive support of the intervention.

#### 4.1.1. Omnibus and Discrete Context

The CPO evaluation model distinguishes the omnibus context and the discrete context, as recommended by Johns [21]. The omnibus context describes the general intervention and implementation setting: occupation (who), location (where), time (when), and rationale (why), whereas the discrete context refers to “specific situational variables that influence behaviour directly or moderate relationships between variables” [21]. According to Johns [21], specific situational variables may comprise task aspects (autonomy, uncertainty, accountability, resources, etc.), social aspects (social density, structure, influence, etc.), and physical aspects (temperature, light, built environment, decor, etc.). Relating to OHI, the omnibus context describes the overall setting in which the OHI takes place independently of the three intervention phases. For instance, it may occur that an intervention at the team-level leads to positive changes in team climate, but these changes do not become apparent because of interfering effects triggered by another project that is implemented at the same time (e.g., introduction of shift work). In this case, if evaluators ignore contextual aspects, such as conflicting projects, they might conclude that the team-level intervention was ineffective; however, it actually had positive effects that were superimposed by another project.

The discrete context refers to specific individual, leader, group, and organisational (IGLO) aspects directly relevant to the implementation and change process. Randall and colleagues [26] further distinguish between two temporal kinds of context: the baseline preintervention context and the context of activated intervention. The CPO evaluation model considers this temporal differentiation by referring to the three above-described phases. In the preparation phase, the discrete context is often evaluated in regard to the question of whether the organisation and its members are ready for the next phase so that the implementation process can gain momentum and flow in the action cycle phase as described in the scenario with the critical line manager (see Section 4.1). In the action cycle phase, the discrete context is most commonly evaluated in regard to the factors that, passively or actively, hindered or promoted the flow of the implementation and change process. In the appropriation phase, the discrete context is evaluated in regard to aspects that will maintain and further develop the induced changes or if they will be reversed.

The differentiation between the two kinds of contexts is important with respect to the extent to which the intervention implementers can influence them. Often, aspects of the omnibus context are hardly or not at all manipulable by intervention implementers, for instance, economic developments. It is nevertheless important to monitor and record the omnibus context for an understanding of the implementation process and an interpretation of intervention outcomes. For example, high dismissal rates caused by an economic crisis (omnibus context) will lead to lower absolute participation rates (implementation process) as a matter of course.

The discrete context, on the other hand, can be changed—not easily, but easier than the omnibus context—and, thus, should be considered as a target of change from the beginning. Due to the close proximity of the discrete context to the implementation process, the discrete context (e.g., leaders' and employees' readiness for change) can have an immediate and stronger influence on the implementation process than the omnibus context.

### 4.2. Process

Referring to Bauer und Jenny [1], process covers “both the implementation processes … and the intended and unintended process of change triggered in organizations and their employees, leading to alterations in …outcomes.” Following this understanding, the CPO evaluation model distinguishes between the implementation process and the change process.

#### 4.2.1. Implementation Process

The CPO evaluation model defines the implementation process as the time-limited enactment of all steps and elements of the original intervention plan. The intervention plan, or so-called overarching intervention architecture, concerns the combination and sequence of single intervention elements that may vary in terms of level (IGLO), target audience (e.g., aging workers and specific departments), type (e.g., workshops, survey, presentations, and practical lessons), implementer (internal or external consultants), and so on. Further, the intervention elements can be arranged in parallel (e.g., if they address different target groups) or in a sequential order (e.g., if they build on each other). There are a range of intervention architectures for OHIs, which usually also incorporate a set of general implementation principles to be followed during the process (cf.  Table 1). For example, during the preparation phase, successful building of a strong coalition of project leaders, defining goals, and raising awareness and commitment are evaluated (all of which will be part of the subsequent discrete context of the action cycle phase). During the action cycle phase, it is evaluated if the sequence and linkage of the intervention elements are implemented as planned, how employees, managers, and other stakeholders perceive the implementation process, and if the intervention successfully shapes a favourable discrete context for the appropriation phase. The latter means that capacities for self-optimisation are built up so that the organisation and its members are capable and willing to further develop the triggers of change processes autonomously. In the case of formative evaluation assignments, the progress of implementation is monitored continuously in order to make adjustments to the original intervention plan if necessary. In the appropriation phase, it is evaluated whether and how the further development, maintenance, and sustainability of the intervention effects are ensured.

For the evaluation of the implementation of single intervention elements, many researchers focus on quantitative indicators such as reach [27] or dose received [17]. But qualitative implementation indicators are also applied [28]. Commonly, researchers apply measures capturing the perceived quality of an intervention element [29, 30], which has proven to be an important factor when doing process evaluation [17, 30]. However, more research is needed on which indicators concerning the implementation of intervention elements are useful and how the appraisal of particular intervention elements influences the overall impact of an intervention.

**Figure 2:**
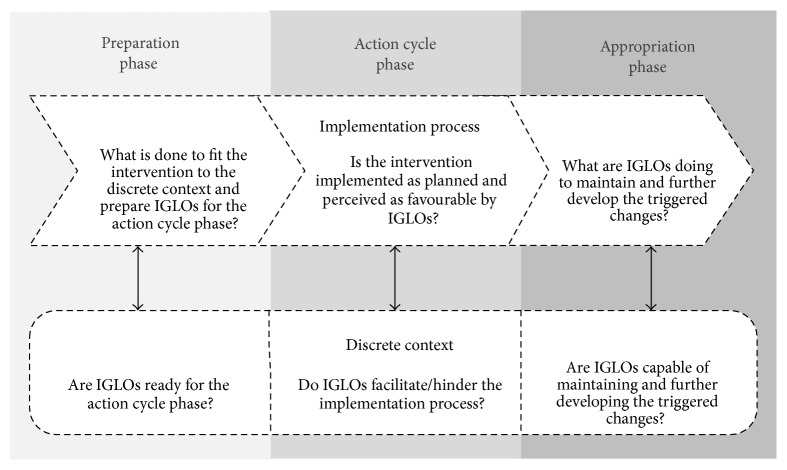
The main questions for evaluating the implementation process and the discrete context with regard to the three intervention phases proposed by the CPO model. IGLO: individual, group, leader, and organisation.

Distinction between aspects of the implementation process and the discrete context is sometimes difficult; thus, in the past, it has often been ignored.  Figure 2 illustrates the main questions for evaluating the implementation process and the discrete context with regard to the three intervention phases.

#### 4.2.2. Change Process

The CPO evaluation model defines the change process as all intended and unintended individual and collective dynamics triggered by the implementation process, leading to alterations in the organisation and its members. Thus, the change process potentially involves all levels of the intervention context from the individual to the organisation (and their environments). As a current overview of OHI approaches shows [1], change processes include, for example, individual and organisational learning, social processes, taking over others' perspectives, realisation of jointly developed action plans for improving work, organisational structure, and strategy.

Regarding the timeline of change, the CPO evaluation model is based on the assumption that the implementation and change processes in OHIs are initiated with the beginning of the preparation phase. During the action cycle phase and the appropriation phase, the change process gains drive and develops its intended dynamics. This is illustrated in Figure 1 by the increasing colour density of the change process arrow. Furthermore, the arrow suggests that the change process does not end with the appropriation phase. The implementation process should trigger changes in the everyday processes and structures in organisations leading to short- and long-term outcomes of interest. Due to the multilevel nature of OHIs, change can spread beyond the single topics (e.g., coping with stress, transformative leadership, and teamwork) or particular target audiences (e.g., single divisions and risk groups) initially addressed by the intervention [31]—leading to unforeseen desirable or undesirable side effects.

Evaluation of the change process can help to reveal what changes were triggered on which level by the implementation process and thus help to better understand the mechanisms of change. There is a lot of research on the different types of change. Authors discuss whether change should be considered as episodic or continuous [32], exceptional or natural [33], but when it comes to the assessment of change, most researchers focus on aspects of the discrete context that influence or shape the change process in a hindering or facilitating way [34, 35]. As the change process is very complex and only partly observable, it still remains as a kind of* black box*. In practice, intervention implementers usually compare baseline and follow-up measures of outcome variables in order to make induced individual and organisational changes visible. Research on valid change process indicators is still scarce, but the psychosocial mechanisms of change described by Karanika-Murray and Biron [36] demonstrate a promising approach to fill this gap (cf.  Table 1). They present “six psychosocial mechanisms that can explain how an intervention can exert its impact on individuals and workgroups: diffusion, sharing, identifying, comparing, influencing and learning” [36]. Evaluating whether, how, and to which extent these mechanisms have evolved, for example, whether and how intervention effects at the individual level have diffused throughout an entire team, might help to make the change process more visible.

### 4.3. Outcomes

Outcome evaluation is mainly concerned with what effects an intervention has had [37]. The CPO evaluation model defines outcomes as all results of the change process that are measurable and at the same time meaningful for the organisation, its members, researchers, and other stakeholders. The CPO model utilises the following three outcome categories: proximate outcomes, intermediate outcomes, and distal outcomes. These categories are based on a trichotomy of outcomes commonly used in the public health community [38–41]. As all outcomes manifest in the intervention context, proximate, intermediate, and distal outcomes can be further observed on all levels of individuals, leaders, groups, and the organisation.

Proximate outcomes, often also labelled as immediate-, initial- or short-term outcomes or first-level targets, refer, for example, to individual skills and collective capacities needed for the change process (i.e., as part of the discrete context) as well as quick-wins in the form of minor but instant structural changes. As such, they can be classified as results of the change process that immediately arise.

Intermediate outcomes, often also labelled as medium-term outcomes or second-level targets, comprise, for example, changes in job demands and resources with regard to factual (job-related) processes such as work load or time pressure and social (people-related) processes such as leadership behaviour or social support [42]. As such, we can define them as results of the change process with regard to factual (job-related) and social (people-related) processes.

Distal outcomes refer to the distal objectives of the interventions such as improved individual health and increased organisational performance, which often depend on change in the intermediate outcomes. These outcomes are often labelled as the overall objectives/goals or, simply, impacts, and we, thus, define them as higher-level results of the change process that evolve over time.

In the literature, most intervention process cycles end up with the evaluation phase measuring the outcome, impact, or effect of the intervention [8, 11, 12]. The CPO evaluation model assumes that changes in outcomes happen continuously as a result of the change process induced by the continuous implementation process; therefore the CPO model depicts evaluation of proximate, intermediate, and distal outcomes in a separate box of the CPO model. Thus, outcome evaluation should be considered not only as an important intervention element where results of the change process are fed back into the organisational system at the end of the intervention, but also as a continuous observation and assessment of change results accompanying the entire implementation and change process, for example, by continuously measuring proximal outcomes to show successful growth in these variables. In this regard, change in proximate outcomes might already be visible after a project information event (preparation phase) or after a particular intervention element, such as a stress management workshop (action cycle phase). Thus, collection and reflection of outcome data are also parts of an intervention and can influence the change process [11].

## 5. Practical Application of the CPO Evaluation Model

### 5.1. Possible Concepts to Specify the Main CPO Categories

The CPO model defines generic categories, which means that they have to be further specified and operationalised by concrete indicators for the purpose of measurement and evaluation.  Table 1 presents a variety of possible concepts and related indicators for these generic categories of the CPO model, considering different intervention approaches and principles. The list is not exhaustive and is mainly compiled on the basis of recent intervention and evaluation research in occupational health psychology [1]. The concrete selection of indicators will depend on the specific intervention theories and logic; interest of stakeholders; and available resources such as budget, time, and manpower. The concepts proposed for evaluating the main CPO categories reveal considerable variance in specification, terminology, and objective—although common themes emerge and could be condensed in future work.

### 5.2. Practical Application of the CPO Model Using the Example of an Evaluation of an OHI at a Hospital in Switzerland

Table 1 presents a list of relatively general concepts to specify the main CPO categories. In order to clarify how the theoretical concepts of the CPO categories can be translated into practice and to show the practical application of the CPO evaluation model in a more concrete way, Table 2 exemplarily illustrates the model's use for the conceptualization of an OHI evaluation by an OHI project that included 31 nursing divisions from a hospital in Switzerland (see Acknowledgments) implemented between 2013 and 2015.

The data collection, data analyses, and the evaluation report of this OHI were structured using the CPO logic. In the first column of Table 2, research questions that might guide the evaluation of an OHI along the CPO categories are presented. The second and third columns show the applied themes/indicators and methods/sources for each CPO category. To better understand the applied themes/indicators and methods/sources, a brief overview of the project with regard to the intervention's architecture, goals, elements, and evaluation instruments is presented in the following paragraph.

The intervention focused on optimizing the working processes in the nursing divisions with regard to lean principles (i.e., reducing waste and enhancing value), fostering interdisciplinary collaboration between nursing and medical staff, strengthening team climate within the divisions, and improving the balance of resources and demands of the nursing teams. A four-day workshop was implemented in each division by the hospital's internal process managers as the main intervention component. A representative selection of employees participated in the workshop; workshop participants consisted of registered nurses at all hierarchical levels, including ward managers. The workshop was evaluated using a paper-based evaluation questionnaire. To evaluate the intervention as a whole, an online survey (comprising items on resources, demands, team climate, work-life balance, etc.) was applied three times at six-month intervals in each division.

The evaluation had a waitlist control group design; that is, the 31 nursing divisions were randomly assigned to either an intervention (*n* = 16) or control group (*n* = 15). For the intervention groups, the workshop took place four to six weeks after the first online survey; for the control group, the workshop took place four to six weeks after the second online survey. Furthermore, four focus group discussions were conducted at the end of the intervention project to gather data on the implementation and change process as well as on the discrete context. The focus group discussion participants were composed as follows: (1) workshop participants who rated the intervention impact positive; (2) workshop participants who rated the intervention impact negative; (3) nonworkshop participants who rated the intervention impact positive; and (4) nonworkshop participants who rated the intervention impact negative.

## 6. Limitations

A thorough evaluation of all CPO categories and subcategories is a difficult endeavour requiring a lot of resources and instruments. In many cases, it will not be possible to evaluate all these aspects due to scarce resources or limited access to information. Furthermore, it can be difficult to specify the CPO categories and develop suitable indicators due to the variety of possible concepts. However, the CPO model at least offers an overview of which categories* could* be evaluated and how these categories could be specified. Depending on the particular intervention project, evaluators can then consciously decide which of these categories should be evaluated and to what extent. It can sometimes be difficult to distinguish between aspects of the implementation process and the discrete context but the questions displayed in Figure 2 may facilitate the distinction of these two categories. The most arguable category of the CPO model is the change process, as it still remains a kind of black box. Consequently, more research is needed on how to assess the change process. Furthermore, the CPO model was only tested in two intervention projects so far. Use of the CPO model across diverse OHIs and evaluation studies may produce a systematic evidence base, building on generic categories for collecting and reporting data. This will help researchers and practitioners to develop more effective and sustainable interventions in the future. Finally, preexisting concepts and indicators have to be tested and compared in different intervention projects in future research in order to identify the critical concepts and develop corresponding valid indicators.

## 7. Conclusions

The CPO evaluation model provides a basis for structured evaluation of combined OHIs in the field by combining context, process, and outcome evaluation. It offers generic evaluation categories and subcategories that are further differentiated in terms of time and hierarchy and displayed by a grid of intervention phases and organisational levels that facilitates detecting changes at different times and levels. Furthermore, it provides a clear taxonomy for a wide range of possible concepts specifying the evaluation categories and subcategories. Development, testing, and selection of concrete indicators need to be realized in future research. However, descriptions of the evaluation of the OHI project at the hospital demonstrate how an evaluation guided and structured by the CPO evaluation model might look. The research questions presented in Table 2 will support intervention researchers in selecting appropriate indicators for a particular intervention project.

In comparison to similar models, the CPO model uses a clearly defined terminology for OHIs, which might facilitate the development of a common language for improving both communication between researchers and company members and the comparability and aggregation of evaluation study results.

Second, it distinguishes the implementation process from the change process. This distinction is essential as it helps differentiate between cause (i.e., the implementation process) and effect (i.e., the triggered change process), which in turn helps researchers and company members understand the mechanics of change. Following Harachi and colleagues [43], it also helps identify the possible causes of intervention failure. In the first instance, the failure may be caused by an implementation failure, which means “that the way the intervention was implemented was incomplete or designed in such a way that the intervention would have failed even if the theory behind the intervention was correct” [7]. Alternatively, the theory/programme failure may be based on false assumptions about how the implementation of the intervention translates into desired outcomes through an assumed change process, meaning “that the theory behind the problem did not address the problem” [7].

Third, it assumes a reciprocal relationship between the implementation process and the discrete context. By doing so, it broadens the hitherto existing understanding of a static context by defining it as a dynamic factor that needs to be systematically considered and that can be transformed during the intervention.

Fourth, it considers outcome evaluation as the continuous observation and assessment of the change results accompanying the entire intervention. Conducting an outcome evaluation continuously from the beginning will help to better understand the dynamics of the change process and to prevent evaluation results from being subject to hindsight biases.

Overall, the CPO evaluation model can serve as a shared mental model throughout the complex intervention evaluation process by supporting organisational members, project leaders, implementers, and evaluators in establishing objectives, selecting possible evaluation concepts from OHI literature, developing key indicators, gathering data, and reporting evaluation results in a structured and succinct way.

## Figures and Tables

**Table 1:** Possible concepts to specify the main CPO categories.

*CPO category* Definition	Selection of possible concepts in the intervention research literature (for concrete indicator examples see corresponding references)
*Omnibus context* General intervention and implementation setting	(i) Occupation (who), location (where), time (when), and rationale (why) [21] (ii) Economic developments, regulatory/trade/economic policies, technological innovations, and changing worker demographics and labour supply (*external context* [44]) (iii) Organizational restructuring, new quality and process management initiatives, alternative employment arrangements, work/life/family programs and flexible work arrangements, and changes in benefit and compensation systems (*organizational context* [44])

*Discrete context* Specific individual, leader, group, and organisational (IGLO) aspects directly relevant to the implementation and change process	*Leaders/individuals* (i) Line manager attitudes, employee readiness, and intervention history [20] (ii) Readiness for/stages of change [45, 46] (iii) Mental models of stakeholders [7, 8] *Groups/organisation* (i) Awareness of norms, diversity, early role clarification, manager availability, and constructive conflicts [47] (ii) Climate and culture, task attributes, social-relational aspects of work, worker roles, and career development (*work context* [44]) (iii) Organisational resources, psychological resources, facilitating and obstructing elements of the design, and organization and management of work [48] (iv) Task characteristics, social characteristics, and physical characteristics [21]

*Implementation process* Time-limited enactment of all steps and elements of the original intervention plan	*Implementation of intervention elements* (i) Recruitment, reach (e.g., number of workshop participants), dose delivered (e.g., number of workshops), dose received (e.g., engagement in workshops), fidelity of implementation as planned, and participants attitudes to and satisfaction with the intervention [16, 17, 27, 30, 49, 50] (ii) Participation in intervention decision, stakeholder appraisals of intervention plans and activities, and observable and perceived exposure to intervention activities [48] *Implementation of intervention architecture* (i) Thorough diagnosis, definition of goals/vision, raising of shared problem awareness, building of coalition of leaders and drivers, lively communication, good time management, professional project organization and responsibilities, empowerment for self-optimisation, quick-wins and motivation, process flexibility, monitoring and controlling, and anchoring of change (*12 success factors of change *[51]) (ii) Multilevel collaboration, active support from managers, explication of tacit knowledge, continuous evaluation and adjustment, visualisation of process and results, appointment of facilitator, and defined project period [52]

*Change process* All intended and unintended individual and collective dynamics triggered by the implementation process, leading to alterations in the organisation and its members	(i) Diffusion, shared meaning making, social identity building, social comparison processes, interpersonal influences, and social learning (*psychosocial mechanisms of change* [36])

*Proximate outcomes* All results of the change process that immediately arise	(i) Minor structural and strategic modifications (e.g., adapted agendas, rules of communication, and well-being checks [53]) (ii) Changes in attitudes, values, and knowledge [8] (iii) Individual competencies and collective capacities for self-optimisation in teams [54]

*Intermediate outcomes* All results of the change process with regard to factual (job-related) and social (people-related) processes	(i) Demand-control-support [55] (ii) Effort-reward-Imbalance [56] (iii) Job demands and resources [57] and ratio of resources and demands [58] (iv) Team climate [59] (v) Healthy organizational resources and practices: task resources, social resources, and healthy practices (*HERO model* [60]) (vi) Collective general resistance resources [61] (vii) Work-related sense of coherence [62]

*Distal outcomes* All higher-level results of the change process that evolve over time	(i) General health, mental health, and vitality (*health and well-being scales of the COPSOQ Questionnaire *[63]) (ii) Healthy employees: efficacy beliefs, trust, positive emotions, resilience, and work engagement/healthy organizational outcomes: organizational commitment, high performance, customer loyalty/satisfaction, and corporate social responsibility (*HERO model* [60]) (iii) Individual and collective sense of coherence [64]

*Note*. CPO: context, process, and outcome.

**Table 2:** Evaluation of an organisational health intervention (OHI) with special focus on lean processes at a hospital in Switzerland.

CPO category and corresponding research question	Themes/indicator	Methods/source
*Omnibus context* In what kind of organisation (size, structure, etc.) is the intervention implemented? How does the political, social, and economic environment look like?	(i) General information on the hospital: location, number of divisions and employees, hierarchical structure, financing, and so forth (ii) Specific information on nursing departments: structure of nursing departments, types of nursing professions, number and characteristics of nursing personnel, and so forth (iii) Current political and economic situation/changes that are relevant for the nursing divisions (iv) Previous intervention projects in the nursing divisions	(i) Discussions with project leaders/head of nursing divisions (ii) Documentary analysis (documents provided by the hospital)

*Discrete context* Are IGLOs ready for the action cycle phase? Do IGLOs facilitate/hinder the implementation process? Are IGLOs capable of maintaining and further developing the triggered changes?	*Leaders/individuals* (i) Project commitment and readiness for change *Groups/organisation* (i) Planned projects/alterations during the intervention duration (ii) Reasons for the intervention project (iii) Provided resources (time and budget), stability of project personnel, and information politics (iv) Openness for novelty, communication culture, and channels	(i) Discussions with project leaders/head of nursing divisions (ii) Four focus group discussions

*Implementation process* What is done to fit the intervention to the discrete context and prepare IGLOs for the action cycle phase? Is the intervention implemented as planned and perceived as favourable by IGLOs? Are capacities for appropriation built up? What are IGLOs doing to maintain and further develop the triggered changes?	*Implementation of intervention elements* (i) Implementation of workshops (a) Number of workshops (b) Number of workshop participants (c) Composition of participants (d) Quality appraisals of workshop (e) Outcome expectancies (f) Satisfaction with measures (developed during the workshop) (g) Output of measures for improving the work situation (ii) Implementation of employee surveys (a) Survey period (b) Reach *Implementation of intervention architecture* (i) Open questions	(i) Intervention planning chart (ii) List of measures (developed during the workshop) (iii) Short questionnaires applied at the second and fourth workshop days (iv) Participation rate in the three-wave survey/information on the team sizes given by the team leaders (v) Focus group discussions

*Change process* Does the implementation trigger a process of change? How do the triggered changes disseminate among IGLOs? How do the triggered changes evolve over time?	(i) Transfer of workshop training and output to the team: communication, actions of workshop participants, and reactions of nonworkshop participants (ii) Visibility of the implementation of measures to nonworkshop participants (iii) Dynamics triggered with regard to interpersonal influences and social learning within the teams	(i) Focus group discussions

*Proximate outcomes* Which effects arise immediately?	(i) Changes concerning waste of resources, efficient use of time, and collaboration	(i) Focus group discussions

*Intermediate outcomes* Which effects arise with regard to factual (job-related) and social (people-related) processes?	(i) Changes in the resource-demands-ratio (ii) Changes in the interprofessional collaboration (iii) Changes in team climate, work organisation, and supervisor behaviour	(i) Three-wave survey (ii) Focus group discussions

*Distal outcomes* Which higher-level effects evolve over time?	(i) Positive psychosocial health (engagement and satisfaction) (ii) Negative psychosocial health (stress symptoms and negative feelings)	(i) Three-wave survey (ii) Focus group discussions

*Note*. CPO: context, process, and outcome; IGLO: individual, group, leader, and organisation.
